# Application of a modified Poisson model in identifying factors associated with prevalence of pregnancy termination among women aged 15 – 49 years in Uganda

**DOI:** 10.4314/ahs.v22i3.12

**Published:** 2022-09

**Authors:** Edson Mwebesa, Mary Nakafeero, David Guwatudde, Nazarius Mbona Tumwesigye

**Affiliations:** 1 Muni University, P.O.Box 725, Arua District, Uganda; 2 Makerere University School of Public Health, P.O Box 7062, Kampala-Uganda

**Keywords:** Abortion, Pregnancy termination, maternal mortality, Poisson, Contraceptive, Radio

## Abstract

**Background:**

Abortion in Uganda is illegal, only permitted when it places the pregnant mother at risk. This study aimed to apply the modified Poisson model in identifying factors associated with the prevalence of pregnancy termination among women of reproductive age in Uganda.

**Methods:**

The 2016 Uganda Demographic Health Survey (UDHS) data were used in this study. More than 18,000 women of the age of 15 – 49 years participated in this study. A modified Poisson model that incorporated sampling weights was used to establish the factors associated with pregnancy termination.

**Results:**

In Uganda, 18,506 (18.1%) had ever had a pregnancy terminated. The results revealed that, the woman's age [APR = 3.15, 95% CI: 2.72–3.63], being married [APR = 1.55, 95% CI: 1.40–1.71], mass media exposure [APR = 1.18, 95% CI: 1.08–1.29], working status [APR = 1.21, 95% CI: 1.09–1.35], and having visited a health facility [APR = 1.20, 95% CI: 1.10–1.31] were positively significantly associated with likelihood of pregnancy termination.

**Conclusion:**

There exists a significant proportion of women who have had their pregnancies terminated in Uganda. It is observed that woman's age, marital status, mass media exposure, having visited a health facility in the last 12 months and working status were main predictors. Based on these results, researchers concluded that the emphasis should be put on improving access to post-abortion care, contraceptive use and media exposure.

## Introduction

Pregnancy termination, otherwise known as induced abortion^1^ or elective abortion and therapeutic abortion is removal of the fetus and placenta or pregnancy tissue from the uterus. In developing countries especially in Africa such as Uganda, induced abortion is unsafe and a major public health issue^2^. Unsafe abortion is defined as “a procedure for terminating an unwanted pregnancy by persons lacking necessary skills or in an environment lacking minimal standards or both”^3^ Globally, about 20 million unsafe abortions occur each year with 95% of these in developing countries and about 67% of maternal deaths are related to these abortions making it the leading cause of maternal death^3^,^4^. Of the abortions that occur, 19 – 20 million abortions are conducted by individuals without the necessary skills, and are thus associated with severe complications such as hemorrhage, sepsis and peritonitis, trauma to the cervix, vagina, uterus and abdominal organs and death^4^–^8^. According to^9^, unsafe abortion explains up to 20% of all deaths during pregnancy. The proportion of unsafe abortions are significantly high in highly restrictive abortion laws regions than with less restrictive law regions^8^ such as Uganda.

Developing countries accounted for approximately 99% of global maternal deaths in 2015 with sub-Saharan Africa (SSA) accounting for 66% of these^10^. This high maternal mortality is attributed to poverty, lack of information about abortion and other maternal health services, distance to the health center, inadequate maternal health services, cultural practices, residence status where women in remote regions do not usually access quality health care. According to^3^, Africa is ranked second in terms of induced abortion rate with 29 occurring per 1000 women aged 15 – 44 years. Almost all the induced abortions are unsafe, with 5.5 million unsafe abortions per year^2^. In East Africa, induced abortion rate is 38 per 1000 women per year^11^.

In Uganda, legal abortion is permitted only for the safety of the woman^7^. This restriction compels the perpetuation of the practice in secrecy and often in unsafe conditions^7^,^12^,^13^. The prevalence of pregnancy termination and factors associated with such prevalence in Uganda are not known. Literature highlights different factors associated with pregnancy termination. Such factors include socio-economic and demographic variables, sexual violence, and interruption of family planning, health seeking behavior, contraceptive use, marital status, and birth order^3^,^4^,^14^,^15^. In this study, the researchers aimed to apply the modified Poisson model in identifying factors associated with the prevalence of pregnancy termination among women of reproductive age in Uganda based on 2016 Demographic Health Survey (DHS) Data.

## Methods

The study used secondary data collected by the Uganda Bureau of Statistics (UBOS) during the 2016 Uganda Demographic Health Survey (UDHS). The sampling frame for this survey was the same as that of the Uganda National Population and Housing Census (NPHC) conducted by UBOS in 2014. The participants in the 2016 UDHS were a random sample selected nationally from 20,880 households, from 15 sub-regions, stratified and selected in two stages. The first stage involved selecting 697 enumeration areas (EAs) of which 162 EAs were selected from urban areas and 535 from rural areas. From the 20,880 selected households, 18,506 women were interviewed. All women aged 15 – 49 years in the selected households were eligible to be part of the study^16^. An approval letter to use DHS data was obtained from the DHS program.

Statistical analysis focused on women of reproductive age 15 – 49 years. The outcome variable was termination of pregnancy, a binary categorical variable, that is, ever had pregnancy terminated or not. The exposure variables considered for this study included age of the mother, marital status, mother's level of education, child's birth order, contraceptive use, frequency of listening to radio, smoking status, and distance to the health facility, among others.

The analysis followed three levels starting with descriptive exploration of the study variables, both the response variable and the predictors. Bivariate analysis level was conducted by performing bivariate modified Poisson regression analysis. Only variables that were associated with pregnancy termination at p-value of 0.20 at bivariate analysis were considered in multi-variable model. Backward elimination technique was applied to remove predictors that were not significant at multi-variable level, one at a time and only variables that were significant were considered as main predictors. Researchers tested for confounding of predictors on age of the woman. Prevalence ratios and their corresponding 95% confidence intervals are presented.

The statistical models for binary outcomes suggested in literature include binary logistic regression model, modified Poisson regression model and log-binomial regression model^17^–^19^ each used based on some definitive features of the outcome variable. The commonest of these regression models is binary logistic model (20) given by

(i) $$\text{logit (}\pi_{\text{i}})=\log\left(\frac{\pi_{\text{i}}}{\text{1}-\pi_{\text{i}}}\right)=\beta_{0}+\beta_{1}X_{1i}+...+\beta_{k}X_{\mathrm{ki}}$$

for *π_i_* = 0 (if subject i have never had a pregnancy terminated) or *π_i_* = 1 (if subject i have ever had a pregnancy terminated) for i = 1,2,...n. *X*_1*i*_ ,..., *β_k_X_ki_* are predictor variables and *π_i_* is the probability of experiencing the outcome. Odds ratio is exp(β). The link function for logistic model is the logit link function. The major weakness of logistic regression is that it tends to over-estimate the risk if the outcome of interest is not rare^20^–^22^.

However, modified Poisson regression model is preferred in cross-sectional studies when the outcome of interest is not rare because it approximates the risk ratios or relative ratios better than binary logistic regression model^17^,^20^,^23^. The link function of modified Poisson regression model is log link. The modified Poisson regression model is the Poisson regression of binomial data using robust error variance (18). Its functional form is in equation (ii) below.


(ii) $$\text{log (}\pi_{\text{i}})=\beta_{0}+\beta_{1}X_{1i}+...+\beta_{k}X_{\mathrm{ki}}$$

where π_i is the probability of experiencing the outcome of interest for subject i, β's is the mean of the ith subject and approximates relative ratios as exp(β).

## Results

A total of 18,506 women participated in this study. Results in Table 1 reveal that 181 in every 1000 women had ever had their pregnancies terminated, that is, 3,346 of the total women who participated in the study. Most of the women were aged between 15 – 24 years 8,086 (43.7%), 11,223 (60.6%) were married, 10,630 (57.4%) had attained at least the primary level of education, 12226 (66.3%) household were headed by males. Most women had given birth to at most two children 9,555 (51.6%), 13,563 (72.2%) live in rural areas and are in high wealth index 8,403 (45.4%), none users of modern method of contraceptive 13,456 (72.7%), exposed to social media (radio, television and newspapers) 14,601 (78.9%). The results also reveal that most of the women had no big problems with distance to health center 11,591 (62.6%), have visited the health facility in the last 12 months 12,692 (68.6%), made decisions on health care with their husbands 4,851 (43.2%), lived in households with at least six members 9327 (50.4%), were working 14,264 (77.2%), did not use cigarettes and tobacco 18,211 (98.4%), belonged to Roman Catholic religion 7,335 (39.6%) and live in central region of Uganda 5,481 (29.6%).

**Table 1:** Demographic Characteristics of Respondents and Prevalence of Pregnancy Termination

Description	Category	Frequency	Percent (%)
Ever had a Terminated Pregnancy (*n* = 18,506)	No	15,160	81.9 [81.2, 82.7]
	Yes	3,346	18.1 [17.3, 18.8]
	15 – 24	8,086	43.7 [42.9, 44.5]
Age groups (Recoded) (*n* = 18,506)	25 – 34	5,595	30.2 [29.4, 31.1]
	35 – 49	4,826	26.1 [25.3, 26.9]
	Single	7,283	39.4 [38.2, 40.5]
Current Marital Status (*n* = 18,506)	Married	11,223	60.6 [59.5, 61.8]
	No education	1,781	9.6 [8.8, 10.5]
Highest level of education (*n* = 18,506)	Primary	10,630	57.4 [55.5, 59.3]
	Secondary and Higher	6,095	32.9 [30.8, 35.2]
Sex of Household head (*n* = 18,506)	Male	12,266	66.3 [65.0, 67.5]
	Female	6,240	33.7 [32.5, 35.0]
	1 – 2 children	9,555	51.6 [50.5, 52.7]
Total children ever born (*n* = 18,505)	3 – 4 children	3,639	19.7 [19.0, 20.4]
	5 and above	5,311	28.7 [27.6, 29.9]
Type of place of residence (*n* = 18,506)	Urban	4,943	26.7 [22.9, 30.9]
	Rural	13,563	73.3 [69.1, 77.1]
Household Wealth Index (*n* = 18,506)	Poor	6,643	35.9 [33.2, 38.7]
	Middle	3,460	18.7 [17.4, 20.1]
	Rich	8,403	45.4 [42.2, 48.7]
Modern Contraceptive Use (*n* = 18,506)	Non user	13,456	72.7 [71.6, 73.8]
	User	5,050	27.3 [26.2, 28.4]
Media use (radio, tv, newspapers and magazines) (*n* = 18,506)	No	3,905	21.1 [19.7, 22.6]
	Yes	14,601	78.9 [77.4, 80.3]
Visited health facility in last 12 months (*n* = 18,506)	No	5,814	31.4 [30.1, 32.8]
	Yes	12,692	68.6 [67.2, 69.9]
Getting medical help for self: distance to health facility (*n* = 18,506)	Big problem	6,915	37.4 [35.3, 39.4]
	Not a big problem	11,591	62.6 [60.6, 64.7]
Woman's working status (*n* = 18,474)	Not working	4,211	22.8 [21.5, 24.1]
	Working	14,264	77.2 [75.9, 78.5]
Does not use cigarettes and tobacco (*n* = 18,506)	No	295	1.6 [1.3, 1.9]
	Yes	18,211	98.4 [98.1, 98.7]
Who decides on health care use (*n* = 11,221)	Woman alone	3,408	30.4 [29.0, 31.8]
	Woman and husband	4,851	43.2 [41.7, 44.8]
	Others	2,962	26.4 [25.0, 27.9]
Number of household members (*n* = 18,506)	1 – 5	9179	49.6 [48.1, 51.1]
	6 and above	9327	50.4 [48.9, 51.9]
Religion (*n* = 18,506)	Anglican	5,774	31.2 [29.6, 32.8]
	Catholic	7,335	39.6 [37.6, 41.7]
	Muslim	2,388	12.9 [11.4, 14.5]
	Others	3,009	16.3 [15.1, 17.5]
Region (*n* = 18,506)	Central	5,481	29.6 [25.5, 34.1]
	Eastern	4,879	26.4 [22.7, 30.4]
	Northern	3,546	19.2 [16.2, 22.5]
	Western	4,600	24.9 [21.5, 28.6]

***Source:*** UDHS Data (2016)

Results from Table 2 indicate associations between different predictors and pregnancy termination from modified Poisson regression model. The results from the multi-variable modified poisson regression model reveal that age of the mother is significantly associated with likelihood of pregnancy termination with [APR = 2.13, 95% CI: 1.86, 2.43] for those aged 25 – 34 years, and [APR = 3.15, 95% CI: 2.72, 3.63] for those aged 35 - 49 years, as compared to those aged 15–24 years. This study shows that the likelihood of pregnancy termination increased with age given the upward trend in the age related-prevalence ratios. With regards to current marital status, the study found out that the likelihood of pregnancy termination was 55% higher among married women than those who were single [APR = 1.55, 95% CI: 1.40, 1.71]. The likelihood of pregnancy termination among women who had secondary or higher level of education was 17% lower in comparison to those who had no education [APR = 0.83, 95% CI: 0.73, 0.94], and among women who had access to media was 18% higher compared to those who were not exposed to mass media [APR = 1.18, 95% CI: 1.09, 1.27]. For women who were not cigarette and tobacco smokers, the likelihood of pregnancy termination was 24% less compared to those who were smokers [APR = 0.76, 95% CI: 0.63, 0.92]. Perceived distance to a health center was negatively associated with the likelihood of pregnancy termination. Women who perceived this distance as not being a big problem, the likelihood of pregnancy termination was 11% less compared to those who perceived the distance as being a big problem [APR = 0.89, 95% CI: 0.83, 0.96]. In addition, the likelihood of pregnancy termination among working women and those who visited the health facility in the last 12 months was 21% [APR = 1.21, 95% CI: 1.09, 1.35] and 20% [APR = 1.20, 95% CI: 1.10, 1.31] respectively, compared to women who are not working and had not visited the health facility in the last 12 months. Geographical region was found to be negatively associated with pregnancy termination. The likelihood of pregnancy termination was [APR = 0.83, 95% CI: 075, 0.91] among those women from Eastern Uganda, [APR = 0.80, 95% CI: 073, 0.89] among women from Northern Uganda, and [APR = 0.62, 95% CI: 0.68] among women from Western Uganda compared to women from central Uganda. Total number of children ever born confounded the relationship between pregnancy termination and age of the mother which was considered a primary exposure in this study.

**Table 2:** Analysis of Factors Associated with Prevalence of Pregnancy Termination in Uganda using UDHS Data 2016

Covariates	Categories	Unadjusted estimates	Adjusted estimates

		UPR (95% CI)	APR (95% CI)
Age groups	15 – 24	1	1
	25 – 34	2.79 (2.51, 3.11) ***	2.13 (1.86, 2.43) ***
	35 – 49	4.21 (3.81, 4.67) ***	3.15 (2.72, 3.63) ***
Current Marital Status	Single	1	1
	Married	2.25 (2.06, 2.47) ***	1.55 (1.40, 1.71) ***
Mother's level of education	No education	1	1
	Primary	0.74 (0.67, 0.81) ***	1.01 (0.92, 1.11)
	Secondary and Higher	0.55 (0.49, 0.61) ***	0.83 (0.73, 0.94) **
Total children ever born	1 – 2 children	1	1
	3 – 4 children	2.16 (1.95, 2.39) ***	1.11 (0.99, 1.26)
	5 and above	2.94 (2.70, 3.20) ***	1.09 (0.96, 1.23)
Type of place of residence	Urban	1	-
	Rural	1.03 (0.94, 1.12)	-
Wealth Index Combined	Poor	1	-
	Middle	1.02 (0.92, 1.12)	-
	Rich	0.99 (0.91, 1.07)	-
Who decides on health care use	Woman alone	1	-
	Woman and husband	0.85 (0.78, 0.93) ***	-
	Others	0.86 (0.78, 0.95) **	-
Modern Contraceptive use	No	1	-
	Yes	1.18 (1.09, 1.27) ***	-
Mass media Exposure	No	1	1
	Yes	1.17 (1.07, 1.28) ***	1.18 (1.08, 1.29) ***
Doesn't use cigarettes & tobacco	No	1	1
	Yes	0.56 (0.46, 0.68) ***	0.76 (0.63, 0.92) **
Distance to health facility	Big problem	1	1
	Not a big problem	0.84 (0.76, 0.91) ***	0.89 (0.83, 0.96) ***
Woman's working status	Not working	1	1
	Working	1.84 (1.65, 2.06) ***	1.21 (1.09, 1.35) ***
Sex of household head	Male	1	-
	Female	0.99 (0.91, 1.07)	-
Visited health facility in last 12 months	No	1	1
	Yes	1.49 (1.37, 1.63) ***	1.20 (1.10, 1.31) ***
Number of household members	1 – 5	1	-
	6 and above	0.96 (0.89, 1.03)	-
Religion	Anglican	1	-
	Catholic	1.05 (0.97, 1.14)	-
	Muslim	1.02 (0.90, 1.16)	-
	Others	0.999 (0.90, 1.12)	-
Region	Central	1	1
	Eastern	0.90 (0.82, 0.99) *	0.83 (0.75, 0.91) ***
	Northern	0.91 (0.82, 1.00) *	0.80 (0.73, 0.89) ***
	Western	0.68 (0.62, 0.76) ***	0.62 (0.56, 0.68) ***
AIC			16754.9
BIC			16880.1

*** p<0.001

** p < 0.01

* p < 0.05

**Source:** UDHS Data (2016)

## Discussion

In this study, it was revealed that the overall prevalence of pregnancy termination was 18.1%. This finding is congruent with the findings in Batu Town, Ethiopia^24^, in Kampala Uganda^25^, in Nepal^26^ and in Ghana^27^ who all found a slightly high prevalence level. However, the findings in Ethiopia^4^, in Hamedan, Iran^28^ and in Mozambique^28^ showed slightly lower prevalence (less than 10%). High prevalence in Uganda can be attributed to low contraceptive use and high rates of unintended pregnancies^7^,^15^.

Significant association was observed between age of the mother and pregnancy termination. It was found out that women aged 35 - 49 years were highly likely to terminate their pregnancies than those aged 15 – 24 years, followed by those aged 25 – 34 years, compared to those aged 15 – 24 years. Similar findings are observed in^4^,^27^–^29^. However, they are in disagreement with the findings of^30^ and^24^. The study also found that marital status, which is, being married (including living with a partner) was statistically associated with pregnancy termination. Studies by 4,30,29, and 27 agree with this finding. However, the findings are not congruent with the findings of 24. This is attributed to low levels of modern contraceptive use among marrieds15 and culture that encourages women to give birth when married or living with a partner.

In the present study, exposure to mass media was found to be significantly associated with pregnancy termination. The study revealed that the odds were higher among those who listen to radio at least once a week. This concurs with finding of^27^ in Ghana and^4^ in Ethiopia.. This might be because women who have access to media get information about where to get maternal health services. The study also revealed that women who do not smoke have lower odds of pregnancy termination compared to those who smoke.

This could be due to the fact that, those who do not smoke likely have healthier lifestyle than those who do. These health lifestyles might also include better health seeking behavior. Regarding distance to the health facility, whether it's a big problem or not while seeking medical help, the odds of pregnancy termination were lower among women who find the distance not a big problem than those who find it a problem. Contrary to this finding,^4^ observed that distance to the health centre being a big problem was insignificantly associated with pregnancy termination. In this study, this could be due to the fact that, when distance to the health facility is not a big problem, health seeking behaviors in terms of family planning might be better, reducing the odds of pregnancy than those who find a big problem.

## Conclusions and Limitations

The prevalence of pregnancy termination in Uganda is 181 per 1000 women. Mother's age, mother level of education, marital status, mass media exposure, distance to the health facility, smoking status, woman's working status, visiting of health facility and region of residence were found to be significantly associated with pregnancy termination. Researchers recommend that emphasis should be put on improving access to post abortion care since there is a strong evidence of pregnancy termination, family planning services, and use of media to sensitize and inform women. However, there are questions of interest that were not asked in this study. Whereas it was found that 181 in every 1000 women had ever had pregnancy terminated, the conditions upon which these pregnancies were terminated are not covered in this study. Whether they were unsafe or safe and the consequences from terminated pregnancies-if unsafe are also not known and cannot be presented in this study.

## Data Availability

The datasets generated and/or analysed during the current study are publicly available in the Demographic Health Survey repository, https://dhsprogram.com/data/available-datasets.cfm.
